# Compact Conformations of Human Protein Disulfide Isomerase

**DOI:** 10.1371/journal.pone.0103472

**Published:** 2014-08-01

**Authors:** Shang Yang, Xi Wang, Lei Cui, Xiang Ding, Lili Niu, Fuquan Yang, Chao Wang, Chih-chen Wang, Jizhong Lou

**Affiliations:** 1 National Laboratory of Biomacromolecules, Institute of Biophysics, Chinese Academy of Sciences, Beijing, China; 2 Laboratory of RNA Biology, Institute of Biophysics, Chinese Academy of Sciences, Beijing, China; 3 Laboratory of Proteomics, Institute of Biophysics, Chinese Academy of Sciences, Beijing, China; 4 University of the Chinese Academy of Sciences, Beijing, China; University of Bergen, Norway

## Abstract

Protein disulfide isomerase (PDI) composed of four thioredoxin-like domains **a**, **b**, **b'**, and **a'**, is a key enzyme catalyzing oxidative protein folding in the endoplasmic reticulum. Large scale molecular dynamics simulations starting from the crystal structures of human PDI (hPDI) in the oxidized and reduced states were performed. The results indicate that hPDI adopts more compact conformations in solution than in the crystal structures, which are stabilized primarily by inter-domain interactions, including the salt bridges between domains **a** and **b'** observed for the first time. A prominent feature of the compact conformations is that the two catalytic domains **a** and **a'** can locate close enough for intra-molecular electron transfer, which was confirmed by the characterization of an intermediate with a disulfide between the two domains. Mutations, which disrupt the inter-domain interactions, lead to decreased reductase activity of hPDI. Our molecular dynamics simulations and biochemical experiments reveal the intrinsic conformational dynamics of hPDI and its biological impact.

## Introduction

**Figure 1:**
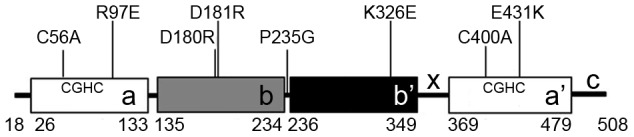
Schematic representations of hPDI and its mutants. The figures show the positions of the domain arrangement of **abb'xa'c**, the active sites (Cys-Gly-His-Cys motif), and point mutations used in this work. The residue numbering is for mature hPDI (after cleavage of the signal sequence).

Disulfide bonds are vital for the stability and function of many secretory and membrane proteins. As a key enzyme in the endoplasmic reticulum, PDI catalyzes the formation, breakage and rearrangement of the disulfides [1], and also functions as a molecular chaperone [2]. PDI is composed of four thioredoxin-like domains in the order of **a**, **b**, **b'**, and **a'**, an **x-**linker between **b'** and **a'**, and a flexible **c** tail (Figure 1) [3]. Domains **a** and **a'** each contains one active site of Cys-Gly-His-Cys motif responsible for thiol-disulfide oxidoreduction [4]. The non-active domain **b'** provides the major substrate binding site [3], [5]. Biochemical studies have indicated that the catalysis of oxidative folding of protein substrates requires all four domains of PDI to function synergically [6].

Although discovered in 1960s, only a few structures of the individual domains of PDI (PDB codes: 1MEK [7], 1X5C, 2BJX [8], 3BJ5 [9] and 2K18 [10]) have been determined in decades. The intrinsic inter-domain flexibility was thought as the primary factor behind the unsuccess of previous trials to crystallize full-length PDI [1]. The crystal structures of yeast PDI (yPDI) at 4°C (PDB code 2B5E) [11] and 22°C (PDB code 3BOA) [12] and hPDI in oxidized (PDB code 4EL1) and reduced (PDB code 4EKZ) states [13] were solved only recently. Significant conformational changes within the enzyme under different environmental conditions were observed indicating high flexibility in the molecular structure of PDI. In yPDI, the **a** arm is more flexible than the **a'** arm and is essential for its enzymatic activity [12]. In contrast, we reported that the C-terminal region (**b'xa'**) of hPDI is more flexible than the N-terminal region (**ab**) and is responsible for its diverse target-binding capacities [14]. Interestingly, in all resolved crystal structures of both yPDI and hPDI, the substrate binding sites were partially occupied by another PDI molecule during crystal packing, which may reflect the binding mode between PDI and substrates [13]. Thus, these structures likely represent different substrate-bound states of PDI with lower flexibility.

The crystallographic studies only provided specific snapshots of various conformations of PDI [11]-[13], while a full understanding of PDI function requires an investigation on its intrinsic dynamics. Recent advances in molecular dynamics (MD) simulations have provided a powerful approach to study the conformational dynamics of a molecule [15]. In this work, using the hPDI structures solved in this lab, we combined MD simulations with biochemical studies to explore the dynamic properties of hPDI and their possible biological impacts. Our simulations suggested that hPDI in solution may adopt more compact conformations which are stabilized mainly by various inter-domain salt bridges. Particularly, the two catalytic domains **a** and **a'** were found to be able to move close enough for a possible intra-molecular electron transfer. Moreover, mutagenesis studies revealed the tight relationships between the compact conformations observed from the MD simulations and the biological activities of the enzyme.

## Materials and Methods

### Molecular dynamics simulations

Crystal structures of hPDI in the oxidized (PDB code 4EL1) and in the reduced (PDB code 4EKZ) states [13] were used as the initial conformations for the MD simulations. Missing regions in one structure were either replaced by the corresponding part in the other structure if they were solved, or modeled with Modloop [16] if they were missing in both structures. The resulting structures containing the residues from Asp^18^ to Ala^478^ of the hPDI sequence were used in simulations. Four systems, oxidized (disulfide bonded) or reduced active sites on each crystallized conformation, were built for the simulations (Table 1). These protein systems were then soaked in 128×96×96 Å^3^ water boxes and neutralized with Na^+^ and Cl^-^. All simulations were performed using the NAMD software package [17] and CHARMM22 all-atom force field [18] with CMAP corrections. In the periodic condition, the particle mesh Ewald summation was used to calculate the electrostatic interactions, and the cut-off for the non-bounded Van der Walls interaction was set to be 12 Å. The energy of each system was minimized via four-step process, i.e., protein heavy atoms fixed, protein backbone atoms fixed, protein Cα atoms fixed and finally all atoms free. The energy-minimized systems were then equilibrated under 1 fs time step at constant temperature at 310 K via Langevin dynamics and constant pressure at 1 atm via the Langevin piston method. The equilibration process contained two steps, 5 ns simulations with Langevin coefficient 5/ps followed by 5 ns simulations with Langevin coefficient 1/ps. The thermal equilibrium of the systems was reached as indicated by the stable root-mean square distance (RMSD) of the rigid portion of each domain in the following simulations (Figure S1). The equilibrated systems were finally simulated for ∼300 ns under 2 fs time step with rigid bond and SETTLE algorithm. The simulation trajectories were analyzed with VMD [19].

**Table 1:** Systems used in MD simulations.

Simulation	Redox state	Initial structure
Sim 1	Oxidized	4EL1
Sim 2	Oxidized	4EKZ
Sim 3	Reduced	4EL1
Sim 4	Reduced	4EKZ

### Protein expression and preparation

The expression plasmids of all hPDI mutants (Figure 1), except that of the isolated domain (**a'c** and **a**) as a generous gift from Dr. Han Cheng in this lab [20], were constructed by overlap extension using the pQE30-hPDI plasmid as a template and verified by DNA sequencing. The resulting proteins, all containing an N-terminal His-tag, were prepared as described previously [21], and stored as aliquots in buffer A (50 mM Tris-HCl, 150 mM NaCl and 2 mM EDTA, pH 7.6) at -80°C. Recombinant human Ero1α was prepared according to Wang, L. *et al* [22]. Protein concentrations were determined by the Bradford method with bovine serum albumin as a standard [23].

### Reductase activity assay

Reductase activity of hPDI and its mutants was determined by monitoring insulin reduction. The reaction was initiated by addition of hPDI proteins to 0.1 M potassium phosphate buffer (pH 7.5) containing 130 µM insulin, 2.5 mM EDTA and 0.1 mM DTT. The absorbance at 650 nm, that represents the light scattering resulted from the aggregation of reduced insulin chains, was immediately recorded on a SHIMADZU UV-2501PC spectrophotometer (Shimadzu) at 25°C. The activity was calculated by the maximal slope of the curve relative to the lag time [24].

### Oxygen consumption assay

All reaction components, except Ero1α, were freshly mixed in 100 mM Tris-HAc buffer containing 50 mM NaCl and 1 mM EDTA (pH 8.0) to a total volume of 0.5 ml in the reaction vessel of the Oxygraph Clark-type oxygen electrode (Hansatech Instruments), and the reaction was initiated by injection of 1 µM Ero1α.

### Mass spectrometry

The protein bands were manually excised from SDS-10% PAGE and divided into two aliquots. After dehydration, the gel plugs were incubated in 25 mM NH_4_HCO_3_ with and without 10 mM DTT respectively, for 45 min at 56°C. Then both samples were alkylated with 40 mM iodoacetamide in 25 mM NH_4_HCO_3_ for 45 min at room temperature in the dark and then digested overnight with trypsin (40 ng for each band) at 37°C. The reactions were terminated by adding trifluoroacetic acid to a final concentration of 1%, and desalted using C18 Zip-Tip microcolumns (Millipore) according to the protocol provided by the manufacturer. The samples were then loaded into the instrument in a crystalline matrix of α-cyano-4-hydroxycinnamic acid (5 mg/ml). Matrix-assisted laser desorption/ionization time-of-flight mass spectrometric detection was achieved using an AXIMA-CFR Plus mass spectrometer from KRATOS Analytical (Shimadzu).

### Interaction of domains a and a'c

The **a** and **a'c** in buffer A were incubated with 10 mM GSSG and 50 nM potassium ferricyanide respectively (oxidation) or with 100 mM DTT (reduction) for 2h at room temperature. The reactions were then exchanged to buffer B (50 mM Tris-HCl, 150 mM NaCl and 2 mM EDTA, pH 7.0) by using a Hitrap Desalting column.

The oxidized and reduced **a** domain of 40 µM was respectively incubated with 40 µM reduced and oxidized **a'c** in buffer B at 25°C. Aliquots of 30 µl from each reaction were removed at different times, quenched by 7.5 µl 5× SDS-loading buffer containing 10 mM 4-acetamido-4-male-imidylstilbene-2,2-disulfonic acid, and analyzed via non-reducing SDS-15% PAGE.

## Results and Discussion

### hPDI can adopt more compact conformations than those in its crystal structures

**Figure 2:**
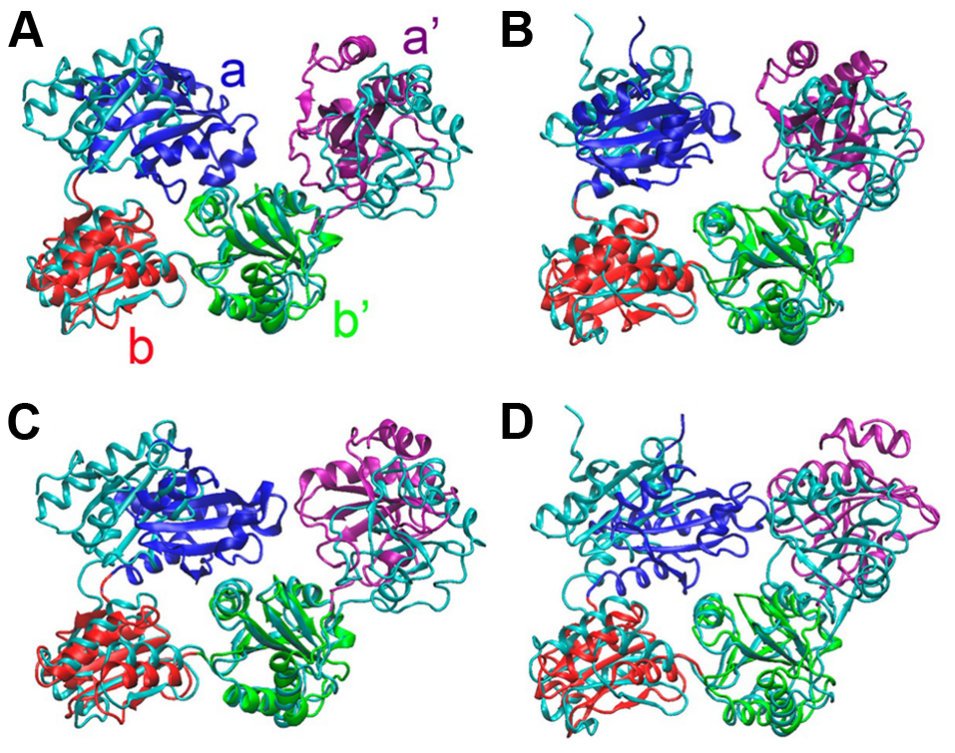
The last snapshots of the molecular dynamic simulations of hPDI. hPDI is composed of four thioredoxin-like domains **a** (blue), **b** (red), **b'** (green) and **a'** (purple). The initial structures of hPDI (PDB code 4EL1 for **A** and **C**; 4EKZ for **B** and **D**) are shown in cyan. Four systems used in simulations were listed in Table 1. The last snapshot of each simulation was denoted as Sim 1 (**A**), Sim 2 (**B**), Sim 3 (**C**), and Sim 4 (**D**).

We carried out four different large scale MD simulations, starting with the solved structures as shown in Table 1, to investigate the intrinsic dynamic properties of hPDI. As the simulation evolves, all four simulations significantly exhibited a tendency to shift away from the corresponding initial structures and change to more compact conformations, which can be represented by the last snapshot of each simulation (Figure 2). The tendency was also clearly observed by the RMSD change of the systems from the initial structures (Figure S2, blue curves) and toward the last snapshots (red curves). In the crystal structures, the space between domains **a** and **a'** was occupied by the adjacent molecule in crystal packing. Since the packing effects are eliminated in the simulations, the observed compact conformations most likely represent the intrinsic stable states of a single hPDI molecule in solution without substrate or partner protein binding. In its resting state, various stable conformations of hPDI may be present in a dynamic equilibrium, including these compact ones observed in our simulations. And these compact forms may have important functional influence.

The compact conformations derived from different simulations are not identical, although the simulations carried out on the oxidized hPDI (Figure 2A, 2B) or reduced hPDI (Figure 2C, 2D) starting from different crystal structures, produce relatively similar conformations. Furthermore, the conformations observed in the simulations on reduced hPDI seem even more compact (Figure 2C, 2D), which is consistent with our crystal structure analysis that the reduced hPDI exists in a closed conformation and the oxidized hPDI in an open conformation [13]. Thus it further supports the proposed intrinsic redox-regulated chaperone activity of hPDI.

In the following in-depth analysis of the simulations we used the geometrical center of the rigid portion of each domain as the center of the domain, because the N- or C-terminal boundary residues of each individual domain are relatively more flexible. The rigid portion of each domain was defined as: **a**, Glu^22^ to Thr^133^; **b**, Thr^138^ to Gln^233^; **b'**, Ile^238^ to Gly^349^; **a'**, Lys^370^ to Glu^471^. The RMSD values of these rigid portions kept at relatively low level (less than 2.5 Å) in all the simulations (Figure S1), indicating that their structural integrities were well maintained during the simulations and may less contribute to the conformational changes of hPDI. Sudden changes in the RMSD values of domain **a'** were observed in the simulations starting from the reduced state (Sim 3 and Sim 4), and detailed analysis on the simulation trajectories indicated that these changes correspond to the conformational changes of the loop Thr^428^ to Phe^440^. The conformations of this loop in the crystal structures of oxidized and reduced hPDI are similar, yet the significance of the conformational change observed in MD simulation is not clear.

**Figure 3:**
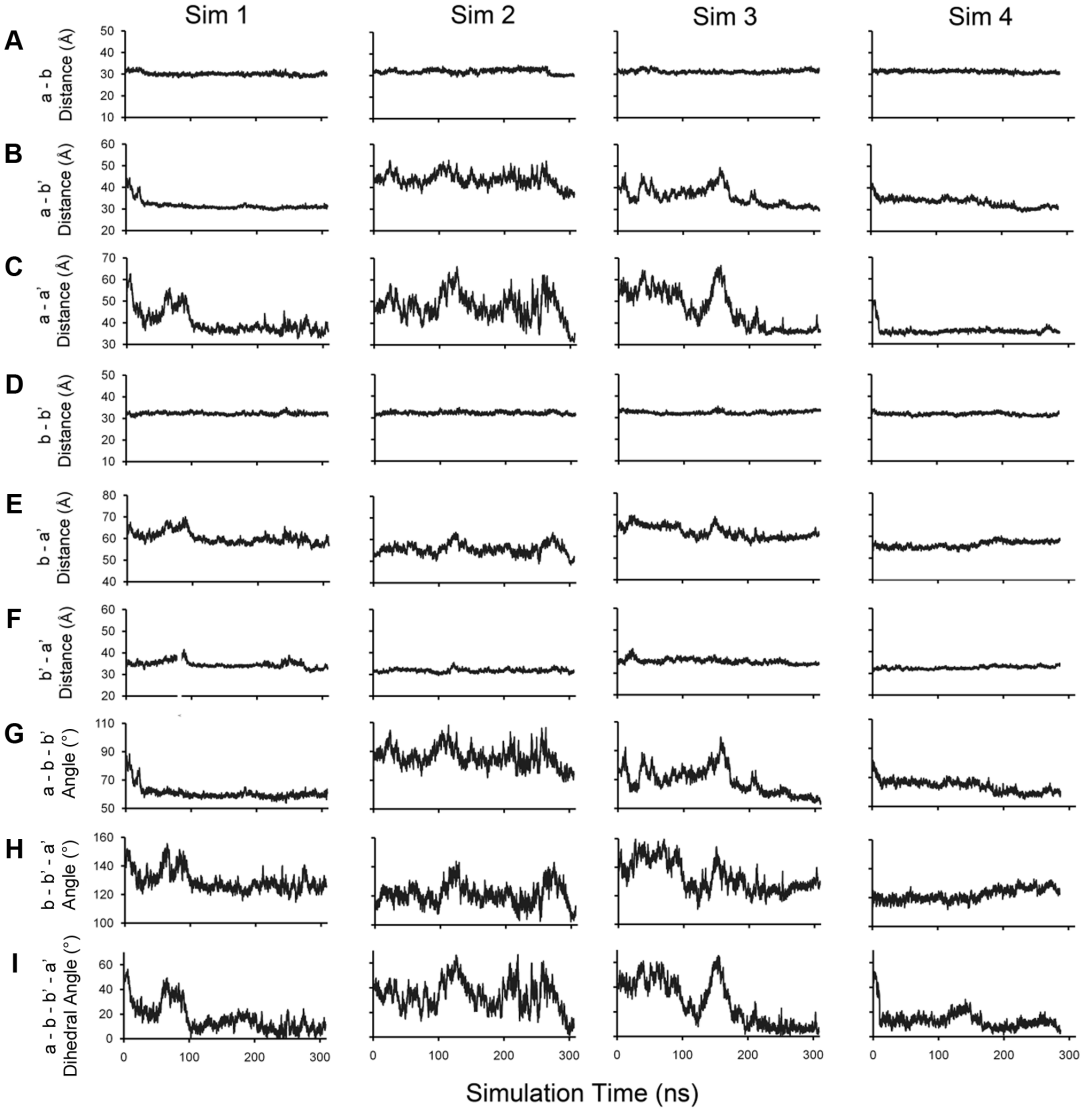
Inter-domain motion of hPDI in the simulations. The time course of the distance between the geometrical centers of domains **a** and **b** (**A**), **a** and **b'** (**B**), **a** and **a'** (**C**), **b** and **b'** (**D**), **b** and **a'** (**E**), **b'** and **a'** (**F**); the angles among domains **a**, **b** and **b'** (**G**), **b**, **b'** and **a'** (**H**); and the dihedral angle between the four domains **a**, **b**, **b'** and **a'** (**I**) were traced for the four simulations as marked. These curves confirm the formation of the compact conformations of hPDI.

In addition, we also observed significant time-dependent changes in the inter-domain distances, the angles among adjacent domains and the dihedral angle of the four domains in the simulations (Figure 3 and Movie S1). The measurements revealed similar features within the compact conformations observed in the different simulations. The distance between domains **a** and **b'** reduces to 32 Å from ∼45 Å in the oxidized and reduced crystal structures (Figure 3B), and the distance between domains **a** and **a'** reduces to ∼35 Å from the original 50∼60 Å (Figure 3C). Simultaneously, the angles among domains **a**, **b** and **b'** markedly reduce about 30∼60° in Sim 1, Sim 3, and Sim 4, and also show a small decrease in Sim 2 (Figure 3G). Moreover, the changes in the dihedral angle among the four domains indicated that their geometrical centers attain nearly coplanar locations (Figure 3I). However, no significant changes in the distance between other adjacent domains (Figure 3A, 3D, 3E and 3F) and in the angle among domains **b**, **b'** and **a'** were observed (Figure 3H). Taken together, these conformational changes of hPDI are most likely to be caused by domain rotation rather than the intrinsic flexibility within each individual domain.

### The compact conformations are stabilized by inter-domain interactions

**Figure 4:**
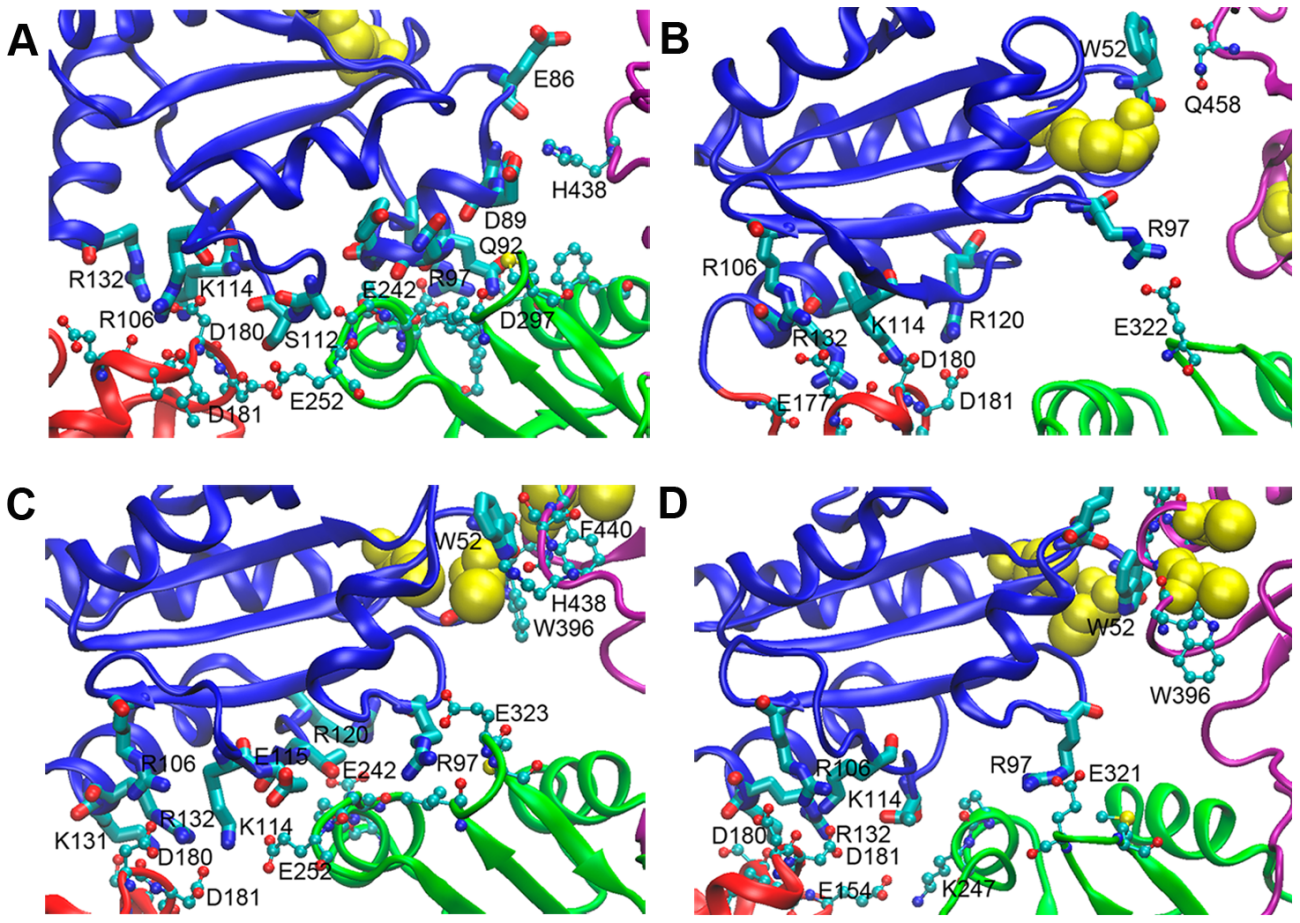
Interactions between domain a and the other three domains of hPDI in the simulations. The last snapshots of Sim 1, Sim 2, Sim 3, and Sim 4 are shown in (**A**), (**B**), (**C**), and (**D**) respectively. Residues in domain **a** are shown using Licorice representation, and residues in other domains are shown as connected balls. The color codes are the same as in Figure 2 with the cysteine residues in the active sites in yellow. (**A**) Sim 1, residue Asp^180^ of **b** forms salt bridges with Lys^114^ of **a**, and is also in the close vicinity to Arg^132^ and Arg^106^ of **a**. Arg^97^ of **a** forms salt bridges with Glu^242^ and Asp^297^ of **b'**; (**B**) Sim 2, Glu^177^, Asp^180^, and Asp^181^ of **b** form salt bridges with Arg^106^, Lys^114^, and Arg^120^ in **a**, respectively. Residue Arg^97^ of **a** forms a salt bridge with Glu^322^ of **b'**; (**C**) Sim 3, Asp^180^ and Asp^181^ of **b** form salt bridges with Arg^106^, Lys^114^, and Arg^132^ of **a**. Arg^97^ of **a** forms a salt bridge with Glu^323^ of **b'**. The active sites of **a** and **a'**, along with residue Trp^52^ and Trp^396^, pack closely with each other; (**D**) Sim 4, the interactions between **a** and other domains are similar as that in Sim 3, except Arg^97^ forms a salt bridge with Glu^321^ instead of Glu^323^.

Once the compact conformations are attained, the systems were found to fluctuate with a relatively small amplitude till the end of the simulations (Figure 3), which suggests the stability of these compact conformations. Since the compact conformations of hPDI were caused mostly by domain rotation, we systematically analyzed the interactions between different domains stabilizing the compact conformations. Relative to the original crystal structures, the **a** domain was observed to exhibit the most significant change during all our simulations (Figure 2). Additionally, extensive inter-domain salt bridge interactions were observed between domains **a** and **b** or **a** and **b'** respectively in the compact conformations (Figure 4, S3). These salt bridge interactions are recognized by the closest distance less than 3 Å of relevant atoms in the positively and negatively charged residue partners. Mutagenesis studies were performed to further validate our findings and to experimentally explore the possible biological significance of the compact conformations of hPDI.

#### (1) Interactions between domains a and b

Aside from the interactions already observed in the reduced crystal structures [13], such as Arg^106^-Glu^177^, Lys^114^-Asp^180^, Arg^132^-Asp^180^, we have identified new electrostatic interactions of Arg^106^ of domain **a** with Asp^180^ of domain **b** (Figure 4A, 4C, and 4D), as well as Lys^114^ (Figure 4C, 4D), Arg^120^ (Figure 4B), and Arg^132^ (Figure 4C, 4D) of domain **a** with Glu^181^ of domain **b**. The time-course dynamics of these salt bridges in each simulation was shown in Figure S3, which indicated that these newly observed salt bridges are dynamic but preferred in the compact conformation. The interactions Lys^177^-Glu^42^ and Lys^177^-Asn^107^ observed in oxidized crystal structure no longer exist in all four simulations. These salt bridges are generally water inaccessible and thus relatively stable. To further elucidate their contributions to the enzymatic activity of hPDI, we prepared a mutant D180R/D181R to partly disrupt the observed interactions between domains **a** and **b**. The reductase activity of the mutant was observed to be attenuated by almost 70% (Table 2) relative to the wild type (WT) hPDI as determined by insulin reduction (Table 2), suggesting the interactions between domains **a** and **b** greatly contribute to the reductase activity of hPDI.

**Table 2:** Reductase activity of hPDI and its mutants.

Protein	Relative activity (%)	Inter-domaininteraction
WT	100	
R97E	68.2 ± 5.7^***^	a and b'
D180R/D181R	26.7 ± 4.5^***^	a and b
K326E	56.0 ± 5.6^***^	b' and a'
E431K	53.6 ± 4.8^***^	b' and a'
K326E/E431K	82.2 ± 7.4^*^	b' and a'
P235G	61.3 ± 2.6^***^	b and b'

The mutants were designed to interfere with inter-domain interactions. The activity was determined according to insulin reduction as described in the text. The activity of WT PDI was taken as 100%. Date were expressed as mean ± S.D. (n = 3). Statistical significance was analyzed by using two-tailed *t*-test, ^***^ and ^*^ represent P<0.001 and P<0.05, respectively.

#### (2) Interactions between domains a and b'

From our simulations with PDI we found that domain **a** may interact directly with **b'**, which has not been reported previously. The interaction is mediated by the salt bridges between oppositely charged residues in domains **a** and **b'** (Figure 4, S3)**.** In several simulated compact conformations, Arg^97^ of domain **a** was found to form salt bridges with various residues of domain **b'**, including the consecutive acidic residues Glu^321^ (Figure 4D), Glu^322^ (Figure 4B) and Glu^323^ (Figure 4C), and Asp^297^ and Glu^242^ on the other side (Figure 4A). None of these electrostatic interactions were reported before, as domain **a** is ∼45 Å away from **b'** in the crystal structures rendering them too distal to have any interaction. These interactions, and those between domains **a** and **b** may make domain **a** less flexible in solution, which is consistent with our previous observation using limited proteolysis [14]. However, the simulation did not show a stable and continuous interaction interface between domains **a** and **b'**, and the salt bridge between Arg^97^ of domain **a** and different residues of **b'** are formed along with the changes of orientation of domains **a** and **b**, suggesting the interactions between domains **a** and **b'** may be dependent on the interactions between domains **a** and **b**.

Mutation of Arg^97^ to glutamate acid, which disrupts the interactions between domains **a** and **b'**, exhibited about 70% reductase activity relative to WT hPDI (Table 2), implying that the interactions between domains **a** and **b'** may also contribute to the reductase activity of hPDI. However, this effect does not seem as robust as that between **a** and **b**, and consistent with the above speculation that interactions between domains **a** and **b'** might be derivation of the interactions between domains **a** and **b**.

**Figure 5:**
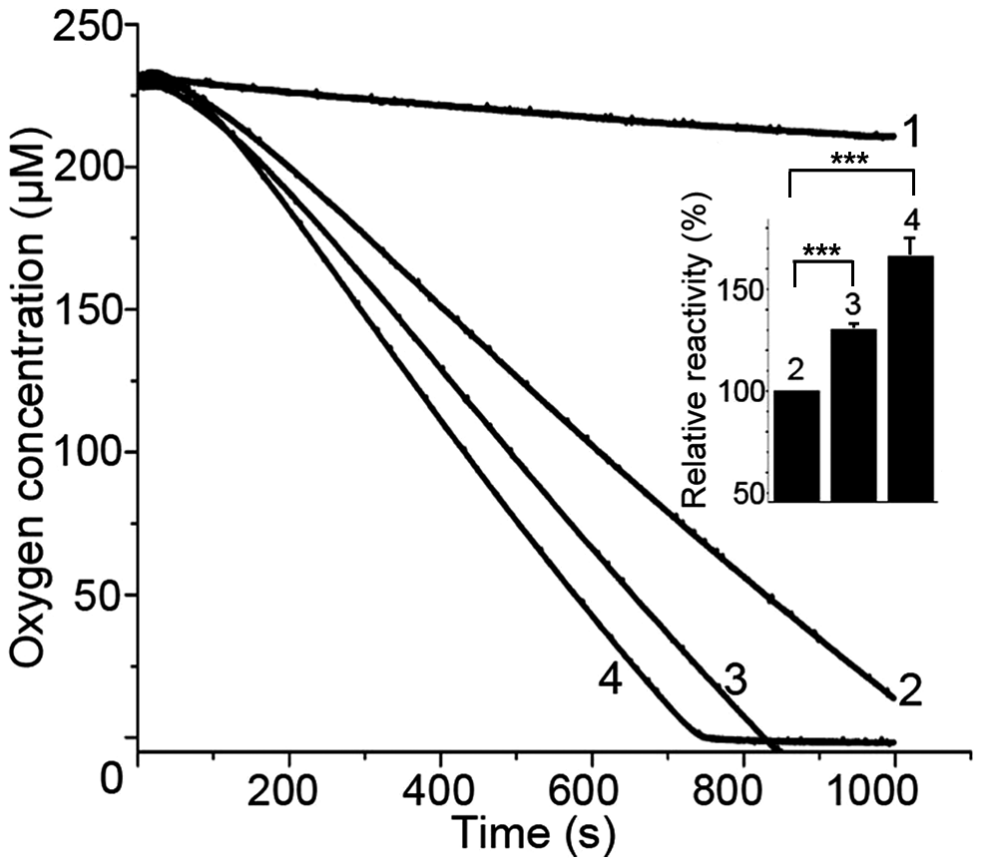
Oxygen consumption in the oxidation reaction of hPDI or its mutants catalyzed by Ero1α. Oxygen consumption was monitored immediately after injection of 1 µM Ero1α into 10 mM GSH without (curve 1) or with 20 µM WT (wild type) hPDI (curve 2), R97E (curve 3), and D180R/D181R (curve 4) at 25°C. Oxidation rate denoted for the reactivity was calculated by measuring the slope of the linear phase of the oxygen consumption curve (deducted the slope of curve 1) with that of WT hPDI as 100%. Date in inset were expressed as mean ± S.D. (n = 3). Statistical significance was analyzed by using two-tailed *t*-test, and ^***^ represents P<0.001.

Surprisingly, in the reaction with hPDI's oxidase Ero1α determined by oxygen consumption, both R97E and D180R/D181R showed higher reactivity than that of WT hPDI (Figure 5). Moreover, the mutant **bb'xa'c** lacking domain **a** also exhibited higher reactivity to Ero1α than the WT hPDI (unpublished data). Domain **a** is believed not to react with Ero1α directly [22], but it can affect the reaction of hPDI with Ero1α through its interaction with **b'**, which suggests a novel role of the **a** domain in regulating the accessibility of substrates or partner proteins to hPDI.

#### (3) Interactions and disulfide transfer between domains a and a'

**Figure 6:**
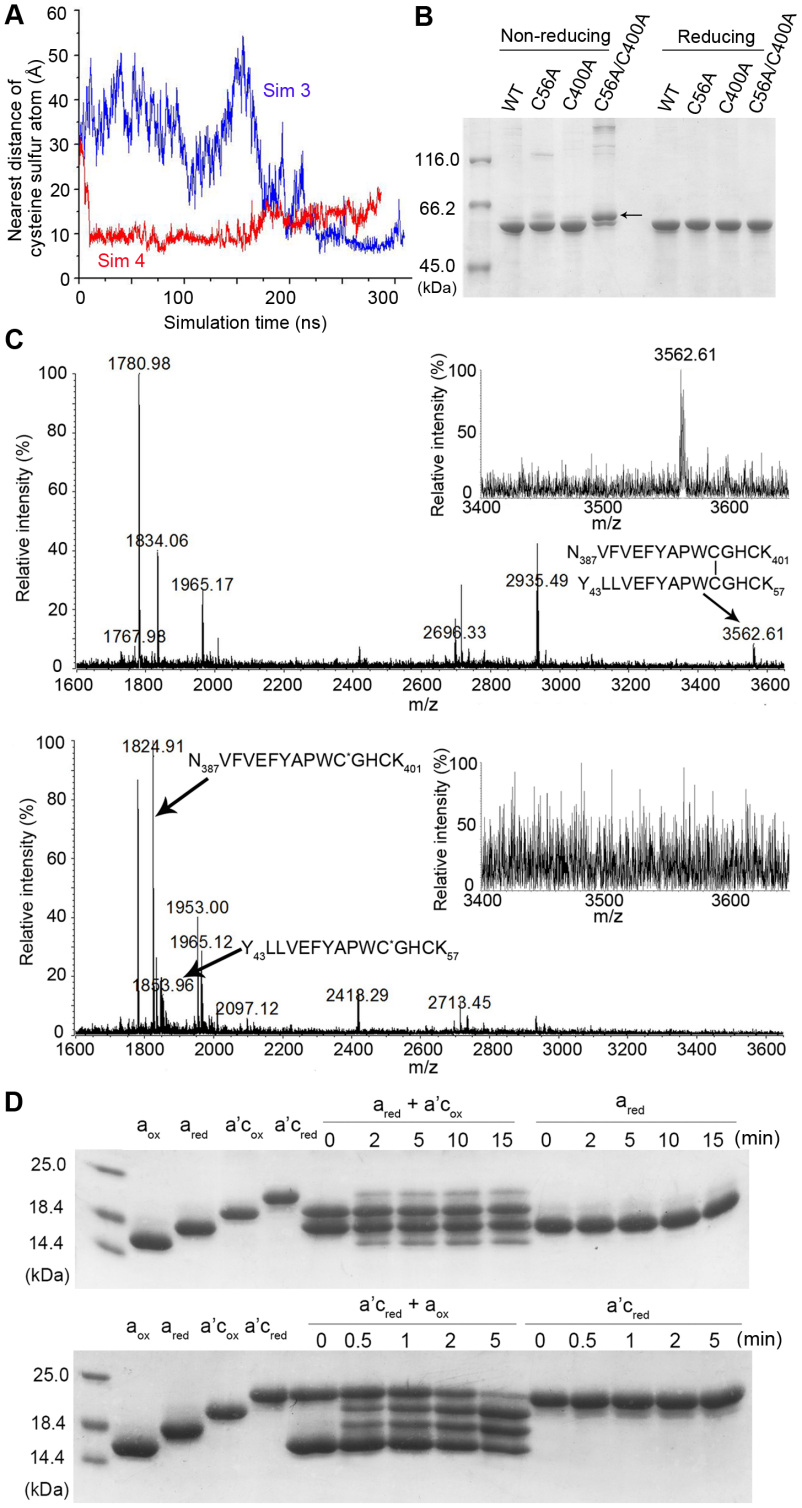
Interactions between the catalytic domains a and a'. (**A**) The time course of the distance between the two active sites of hPDI in Sim 3 (blue) and Sim 4 (red). This distance was measured as the nearest distance between the sulfur atoms of reactive cysteine residues in domains **a** and **a'** respectively**.** (**B**) Redox state analysis of hPDI and its trapping mutants. hPDI and its mutants of 10 µM were alkylated with 10 mM N-ethylmaleimide at room temperature for 15 min to block free thiols, and then analyzed by non-reducing (lanes 1–4) and reducing (lanes 5–8) SDS-10% PAGE. The arrow denotes the extra band in C56A/C400A (lane 4). (**C**) Mass spectrometric analysis of the extra band in (**B**) in the absence (upper) and presence (lower) of 10 mM DTT. The peptides Tyr^43^-Lys^57^ and Asn^387^-lys^401^ and their disulfide-linked form were indicated by arrows. Inset: enlarged mass spectrum profile in the mass range 3400-3650 m/z. C^*^ indicates carboxyamidation form of cysteine. (**D**) Thiol-disulfide exchange reactions between domains **a** and **a'c**. Oxidization of reduced **a** in the presence or absence of oxidized **a'c** (upper). Oxidization of reduced **a'c** in the presence or absence of oxidized **a** (lower). The reactions were quenched by 4-acetamido-4-male-imidylstilbene-2,2-disulfonic acid at the indicated times and analyzed by non-reducing SDS-15% PAGE.

Although Ero1α preferentially oxidizes the active site in domain **a'** [22], the active site in domain **a** was reported to be partially oxidized in the reaction of hPDI with Ero1α [25]. A more recent report showed that the net oxidation rate of hPDI can be altered by changing the reduction potential of the **a** domain, which was dependent on the existence of the intact active site in domain **a'** [26]. Thus an intra-molecular electron transfer between domains **a** and **a'** in the Ero1/PDI pathway was hypothesized. According to the crystal structures [13], the distance between the two active sites in domains **a** and **a'** was determined to be 40.3 Å in the oxidized state and 27.6 Å in the reduced state. The minimum distance between these two active sites so far reported was 16 Å using crosslinking technique [27], still too far for electron transfer. In the present simulations we unexpectedly observed that this distance can decrease to as low as 5.4 Å (Figure 6A and Movie S1). Thus we tried to capture the intermediates in the proposed disulfide exchange reaction between domains **a** and **a'** by using three trapping mutants, C56A, C400A and C56A/C400A. These mutants showed the same mobility on reducing SDS-PAGE, but under non-reducing conditions a thick extra band appeared solely in C56A/C400A (Figure 6B), which was expected to be the intra-molecular Cys^53^-Cys^397^ disulfide-bonded molecule. The extra band was identified by mass spectrometry following tryptic digestion. A signal with a mass (m/z) of 3562.6 (Figure 6C) was detected and perfectly matched with the disulfide-linked Tyr^43^-Lys^57^ and Asn^387^-Lys^401^ peptide (exactly the same mass with the sum of the two peptides minus 2 Da). Under the reducing conditions this signal disappeared but with two new peptide signals instead. The m/z signals at 1853.9 and 1824.9 were 57 Da larger than the mass of Tyr^43^-Lys^57^ and Asn^387^-Lys^401^ respectively, due to cysteine alkylation. The above mass spectrometry data clearly demonstrates the existence of a disulfide between Cys^53^ and Cys^397^ in the C56A/C400A mutant, which provides prima facie evidence that domains **a** and **a'** in an hPDI molecule can move close enough to facilitate electron transfer.

Next, we characterized the disulfide exchange reaction between the active sites in the isolated domains **a** and **a'c**. As shown in Figure 6D, in the absence of oxidized **a'c**, domain **a** alone remained in reduced state for 15 min; in the presence of oxidized **a'c**, two extra bands corresponding to the oxidized domain **a** and reduced **a'c** appeared after 2 min, indicating the oxidation of the reduced **a** domain by oxidized **a'c**, which became reduced at the same time. At 15 min, only ∼15% of domain **a** was oxidized by **a'c**. In this respect, a small fraction of domain **a** in full-length PDI was reported to be also oxidized in the presence of Ero1α [25]. Notably, the oxidation of reduced **a'c** by oxidized **a** is much more efficient, as the fraction of oxidized **a'c** appeared after only 30 seconds, and achieved ∼70% in 5 min. Although domains **a** and **a'** share high sequence identity and very similar reduction potentials [25], their reactivity appears to be different. This could be due to the lower conformational stability of the oxidized **a'** domain, which was reported to be partly unfolded upon the formation of disulfide bond in the active site [4]. The inefficient oxidation of domain **a** by domain **a'** may keep the active site in domain **a** partially in the reduced state, which is required for performing isomerase and reductase activity of hPDI. At present, whether domain **a'** can be intra-molecularly oxidized by domain **a** and the physiological impacts, if any, still need require further study.

#### (4) Interactions between domains b' and a'

**Figure 7:**
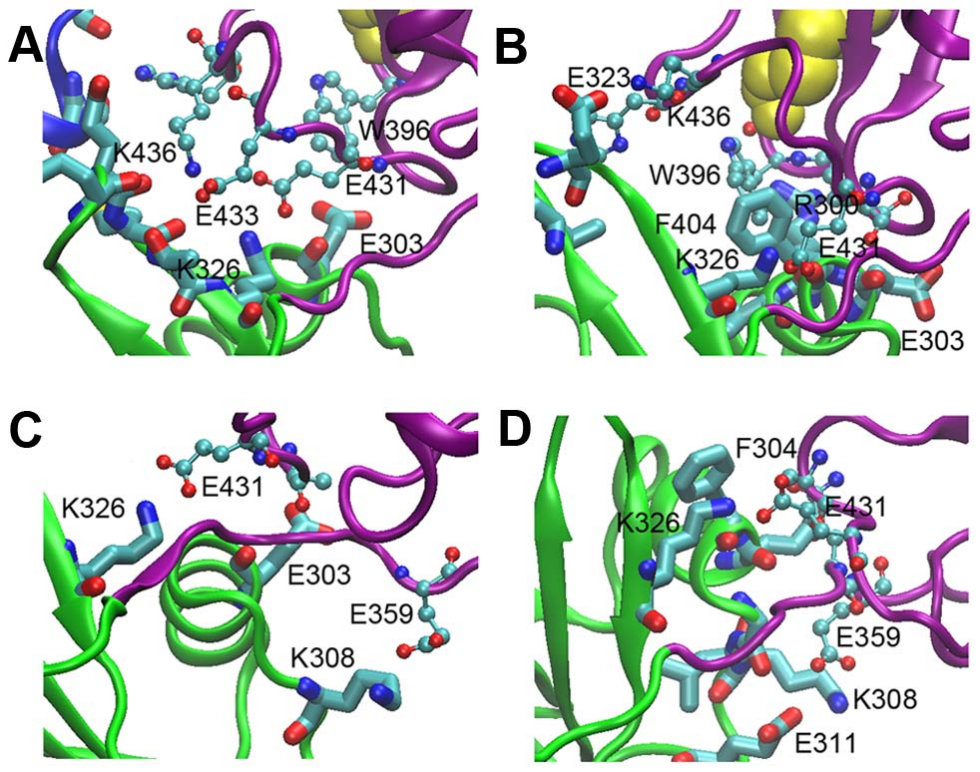
Interactions between domains b' and a' of hPDI in the simulations. The last snapshots of Sim 1, Sim 2, Sim 3, and Sim 4 are shown in (**A**), (**B**), (**C**), and (**D**) respectively. The color codes are the same as in Figure 2 with the cysteine residues in the active site in yellow. Residues in domain **b'** and **a'** are respectively shown in Licorice and connected balls. Salt bridge between Lys^326^ in **b'** and Glu^431^ in **a'** is well maintained, where another new salt bridge between Lys^308^ and Glu^359^ also dynamically formed in (**C**) and (**D**).

The C-terminal region of hPDI molecule, **b'xa'**, has been found to be the minimum redox-regulated cassette [28] and the minimum element for binding to its oxidase, Ero1 [22]. The salt bridge between Lys^326^ of **b'** and Glu^431^ of **a'** and the cation-π interaction between Arg^300^ of **b'** and Trp^396^ of **a'** were observed in the reduced hPDI structure, while both were disrupted in the oxidized structure [13]. Notably, the salt bridge between Lys^326^ and Glu^431^ was consistently observed in the compact conformations obtained from all our simulations (Figure 7, S4), suggesting that the K326/E431 salt bridge might be a key interaction in the stabilization of the contact between domains **b'** and **a'**. Additionally, a new salt bridge between Lys^308^ of **b'** and Glu^359^ of the **x**-linker was found in Sim 3 and Sim 4 (Figure 7C, 7D and S4), which might further contribute to the stabilization of these conformations. As shown in Figure S4, all these salt bridges are dynamic but preferred in the compact conformation. However, contrary to the crystal structure, the Arg^300^-Trp^396^ cation-π interaction was no longer observed in the compact conformations, suggesting that it may only play a role in the change of the redox state of hPDI caused by interaction with its substrates or partner proteins.

To confirm the impact of K326/E431 salt bridge, we prepared K326E and E431K mutants to further elucidate the effects of this salt bridge on the enzymatic activity of hPDI. Compared to WT hPDI, these mutants exhibited an almost half decrease in reductase activity. The mutant K326E/E431K, with a rebuilt salt bridge, restored the activity to 80% (Table 2). The above results strongly support that the interactions between domains **b'** and **a'** are important for the proper conformation and enzymatic activity of hPDI.

#### (5) Inter-domain flexibility of domains b and b'

It has been suggested that the **b** and **b'** domains in yPDI form a rigid base with domains **a** and **a'** as two flexible arms [12]. When the reduced and oxidized hPDI crystal structures are superimposed on domain **b**, domain **b'** shows ∼20° rotation with respect to **b**, indicating a relative movement between **b** and **b'** [13], which was further validated by our simulations by the large value and fluctuation of the RMSD of one domain when the other was structurally aligned (Figure S5). Thus the **bb'** base of hPDI is not as rigid as in yPDI, rather, it displays significant flexibility, similar to ERp27 [29]. The flexibility of the **bb'** inter-domain hinge may be required to regulate the interactions of hPDI with different substrates by adopting alternative orientations.

Since Pro^235^ is the only residue between domains **b** and **b'** and is conserved in mammals, we speculated on its possible role in the inter-domain motion of **bb'**. However, the P235G mutant, which was expected to enhance the mobility between **b** and **b'**, showed only 60% of the reductase activity of WT hPDI (Table 2), suggesting that the limited rigidity of **bb'** may be required for the reductase activity of hPDI.

Our long time scale MD simulation studies reveal more compact conformations of hPDI with its active sites more proximal than those observed in the crystal structures, indicating significant molecular flexibility, which is important for the function of hPDI as shown by our mutagenesis studies. Our detailed analyses of the inter-domain interactions indicate that the compact conformations are formed mainly by domain motions and stabilized by inter-domain interactions, particularly water inaccessible salt bridges. The solved crystal structures probably show the substrate-bound state, and the compact conformations observed by MD simulations likely represent its resting state, which play roles in substrate recognition and/or substrate release during the catalytic cycle.

We have provided first evidence for a possible intra-molecular electron transfer within an hPDI molecule by characterization of the hPDI intermediate with a disulfide between domains **a** and **a'**, as well as the dynamic explanations by MD simulations. The fact that domains **a** and **a'** can locate close enough also arise new insights into the possible synergy of the two active sites of hPDI during its enzymatic reactions.

Our MD simulation of hPDI has shed more light on understanding the contributions of flexible and dynamic conformations of hPDI to its functions. hPDI in the resting state may exist in dynamic equilibrium with various stable conformations, including the conformations observed in crystal structures and the compact conformations indicated by the simulations. This dynamic nature is important for the biological functions of hPDI, as the different conformational states of hPDI allows accommodating substrates with different shapes, sizes, or folding extents and partner proteins with different activities.

## Supporting Information

Figure S1 **RMSD of each domain of hPDI during MD simulations.** The rigid portion of each domain was defined in the text and shown with the color as indicated in the inset of Sim 1. All the four rigid portions remained stable for the RMSD kept at low level (less than 2.5 Å).
(TIF)

Figure S2 **RMSD of the hPDI molecule during MD simulations compared with the corresponding initial conformation (blue) and with the last snapshot of the simulations (red).**
(TIF)

Figure S3 **The time course of the closest distance between the residue pairs involved in the salt bridges between domains a and b or b'.** The closest distance are the minimal distance between all acceptor and doner atoms of the two residues. The threshold to define the salt bridge is 3 Å (black dashed lines).
(TIF)

Figure S4 **The time course of the closest distance between the residue pairs involved in the salt bridges between domains b' and a'.** The black dashed lines are the 3 Å threshold to define the salt bridge.
(TIF)

Figure S5 **Domains b and b' of hPDI undergo relative movement.** The time course of RMSD of domains **b** (blue) and **b'** (red) when domain **b** (**A**) or **b'** (**B**) is structurally aligned with the corresponding crystal structure.
(TIF)

Movie S1 **Representative movements of the domains of hPDI in Sim 4.** With the **b** domain structurally aligned to the crystal structure, it is clearly shown that domains **a** and **a'** become much closer and the overall conformation becomes more compact as time evolves. Cysteine residues at the active sites are indicated as sticks with the sulfur atoms in yellow.
(MPG)
