# Supplementary figures and images for: Compact Conformations of Human Protein Disulfide Isomerase
Source: PLoS One. 2014 Aug 1;9(8):e103472. doi: 10.1371/journal.pone.0103472 (PMC4118876; doi:10.1371/journal.pone.0103472)

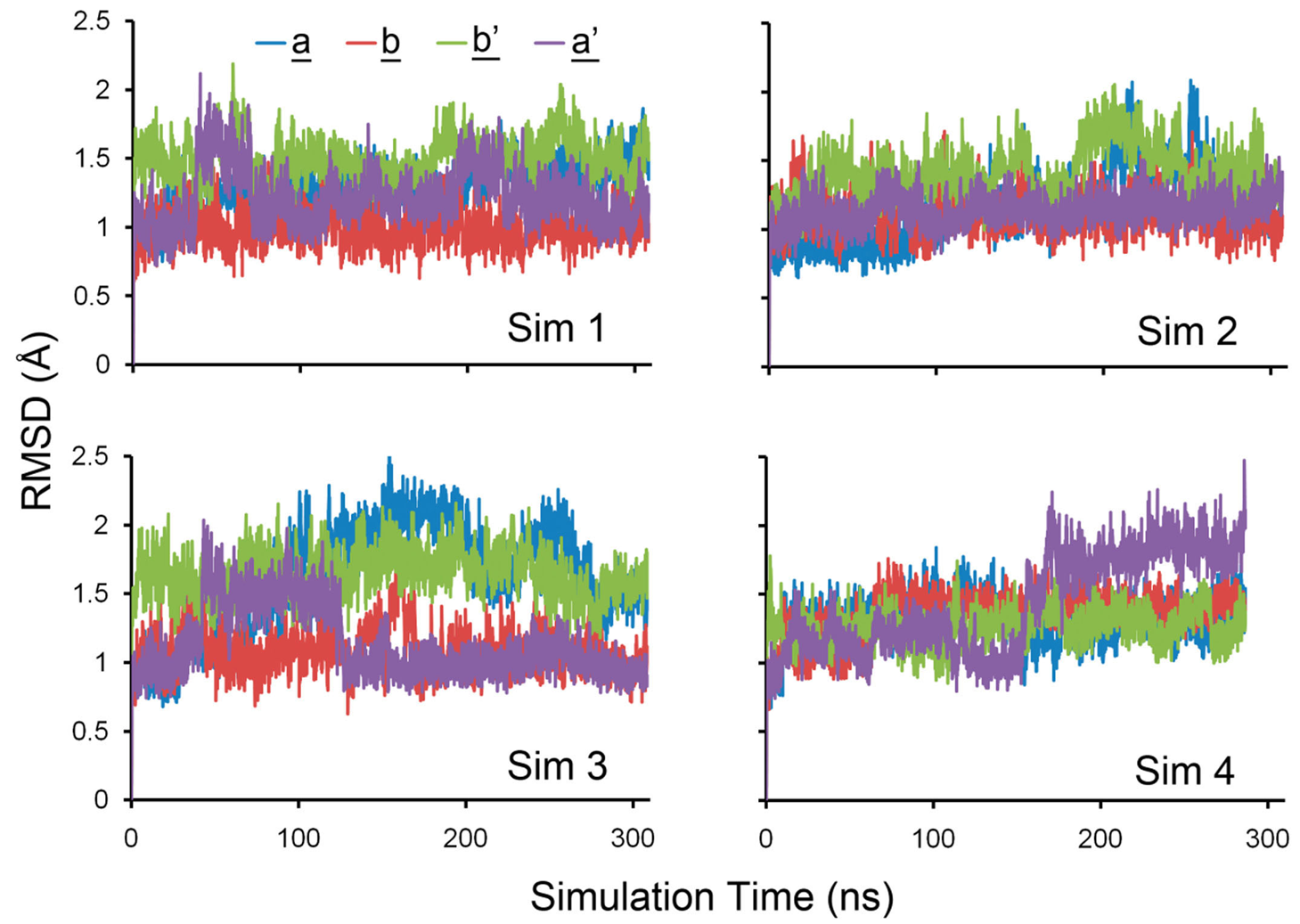

Supplement: Figure S1 — RMSD of each domain of hPDI during MD simulations. The rigid portion of each domain was defined in the text and shown with the color as indicated in the inset of Sim 1. All the four rigid portions remained stable for the RMSD kept at low level (less than 2.5 Å). (TIF) [file pone.0103472.s001.tif]

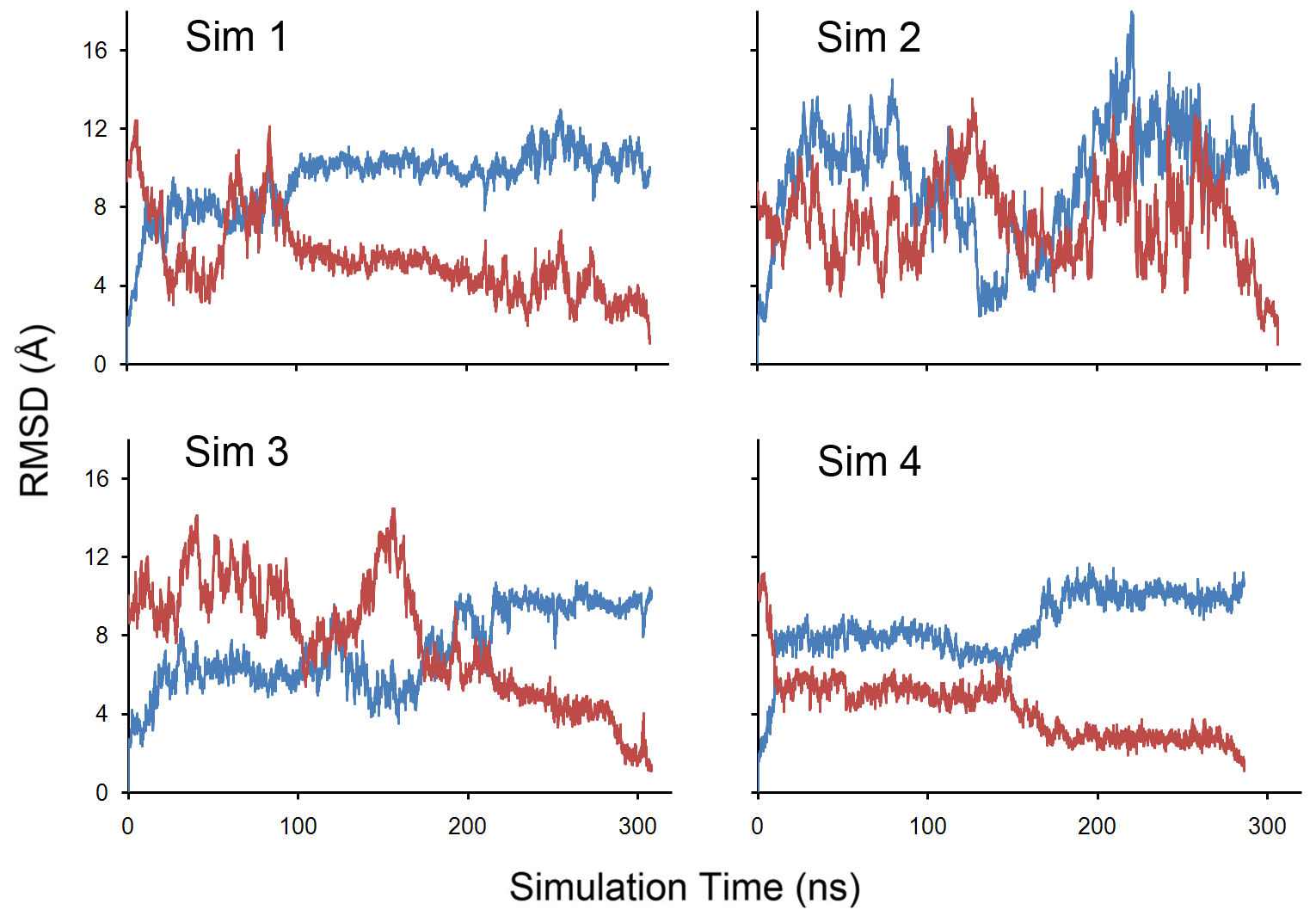

Supplement: Figure S2 — RMSD of the hPDI molecule during MD simulations compared with the corresponding initial conformation (blue) and with the last snapshot of the simulations (red). (TIF) [file pone.0103472.s002.tif]

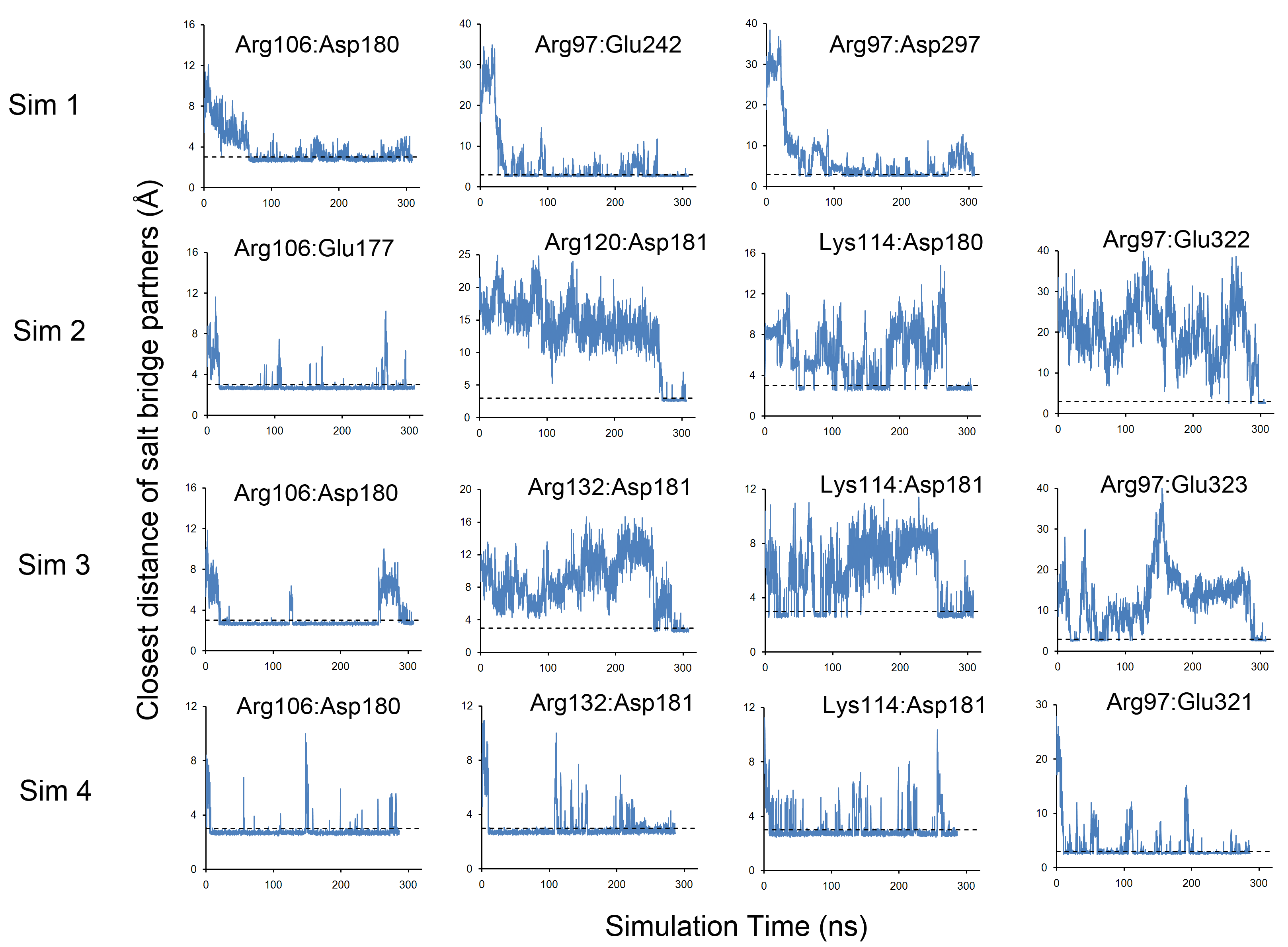

Supplement: Figure S3 — The time course of the closest distance between the residue pairs involved in the salt bridges between domains a and b or b'. The closest distance are the minimal distance between all acceptor and doner atoms of the two residues. The threshold to define the salt bridge is 3 Å (black dashed lines). (TIF) [file pone.0103472.s003.tif]

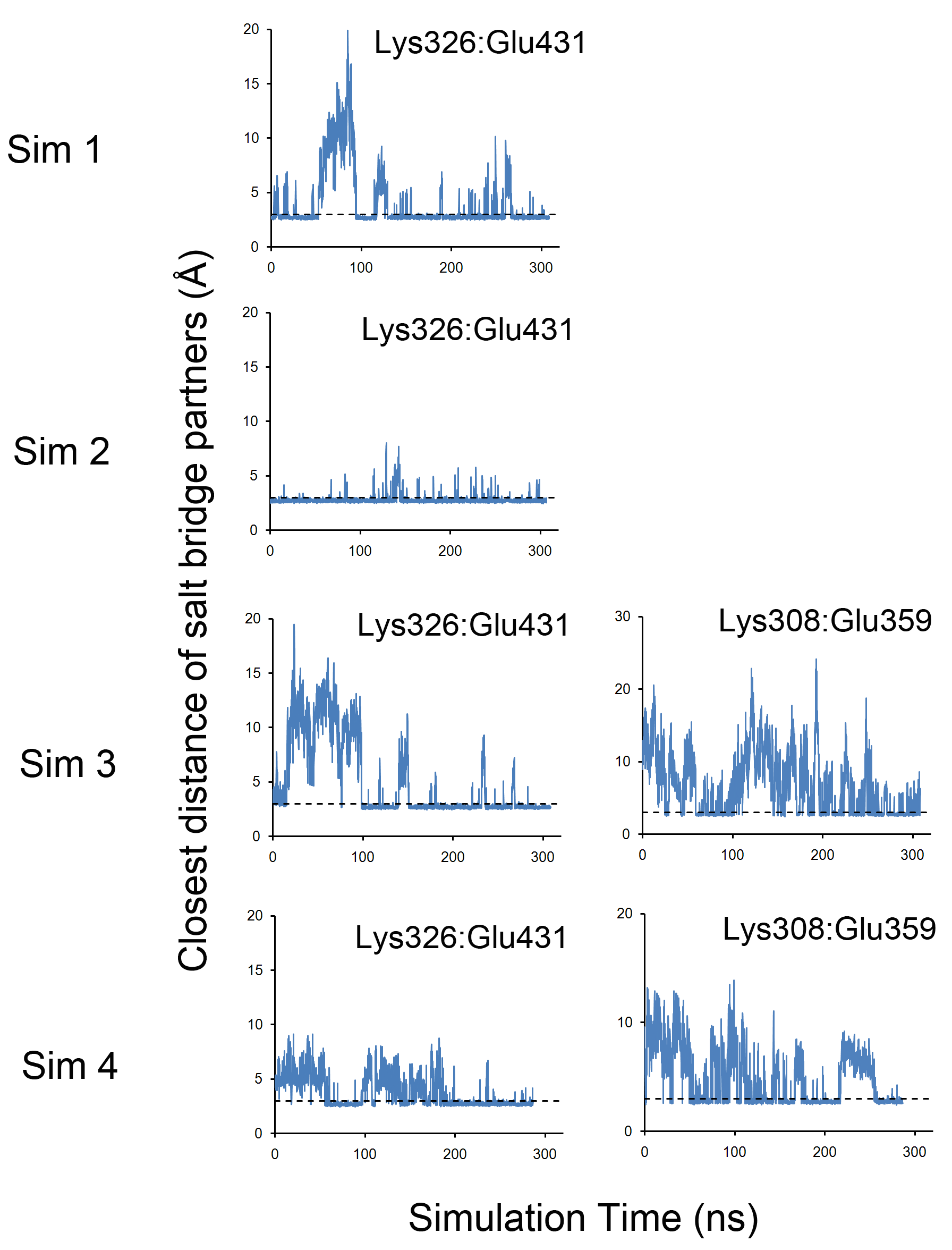

Supplement: Figure S4 — The time course of the closest distance between the residue pairs involved in the salt bridges between domains b' and a'. The black dashed lines are the 3 Å threshold to define the salt bridge. (TIF) [file pone.0103472.s004.tif]

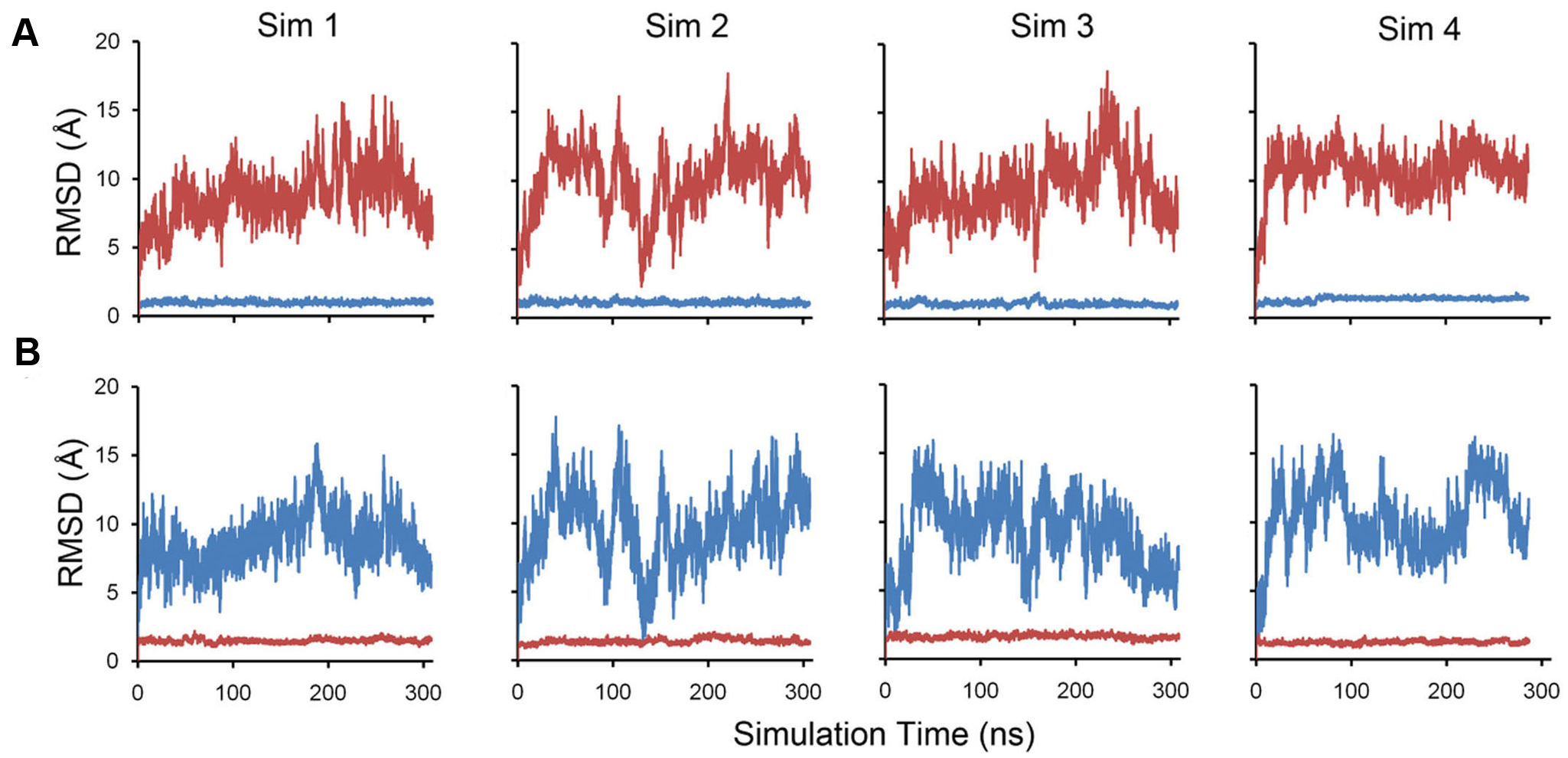

Supplement: Figure S5 — Domains b and b' of hPDI undergo relative movement. The time course of RMSD of domains b (blue) and b' (red) when domain b (A) or b' (B) is structurally aligned with the corresponding crystal structure. (TIF) [file pone.0103472.s005.tif]
